# Severe Pulmonary Suppuration with Infection-Induced Systemic Inflammatory Response Syndrome following Tongue Cancer Surgery in a Patient Undergoing Tocilizumab Therapy for Rheumatoid Arthritis

**DOI:** 10.1155/2014/649086

**Published:** 2014-04-29

**Authors:** Kenji Yamagata, Nobutake Shimojo, Hiroyuki Ito, Junya Ijima, Shogo Hasegawa, Toru Yanagawa, Taro Mizutani, Hiroki Bukawa

**Affiliations:** ^1^Department of Oral and Maxillofacial Surgery, Faculty of Medicine, University of Tsukuba, 1-1-1 Tennodai, Tsukuba, Ibaraki 305-8575, Japan; ^2^Department of Emergency and Critical Care, Faculty of Medicine, University of Tsukuba, 1-1-1 Tennodai, Tsukuba, Ibaraki 305-8575, Japan

## Abstract

A 65-year-old woman with rheumatoid arthritis treated by tocilizumab (TCZ) presented with tongue squamous cell carcinoma. While surgery was performed without any complications the aspiration pneumonia rapidly worsened by postoperative day 2 and severe pulmonary suppuration in the right lung field with infection-induced systemic inflammatory response syndrome (SIRS) was diagnosed. Antibiotic and respirator treatment improved her condition. The anti-inflammatory effect of TCZ may mask the symptoms and signs of severe infection with SIRS.

## 1. Introduction

Although the etiologies of rheumatoid arthritis (RA) are not fully understood, proinflammatory cytokines such as tumor necrosis factor- (TNF-) *α*, interleukin- (IL-) 1, and IL-6 have been shown to play a role in the pathology of RA and, as such, are potential therapeutic targets [1]. Tocilizumab (TCZ) is a humanized anti-IL-6 receptor monoclonal antibody that binds to both circulating soluble IL-6 receptor and membrane-bound IL-6 receptors, thereby inhibiting IL-6 binding to both forms of its receptor [2]. Because IL-6 has a pivotal role in the host defense reaction against microorganisms [3], patients treated with TCZ should be closely monitored for signs of infection. Pneumonia is a common problem in head and neck cancer (HANC) postsurgical patients [4]. A search of the literature revealed only four cases of organizing pneumonia induced by TCZ [5–7], while no account of aspiration pneumonia following HANC surgery could be found. We describe here the first case of severe pulmonary suppuration from aspiration pneumonia complicated by infection-induced systemic inflammatory response syndrome (SIRS) in a postsurgical patient with tongue cancer who had received TCZ treatment for RA.

## 2. Case Report

**Figure 1:**
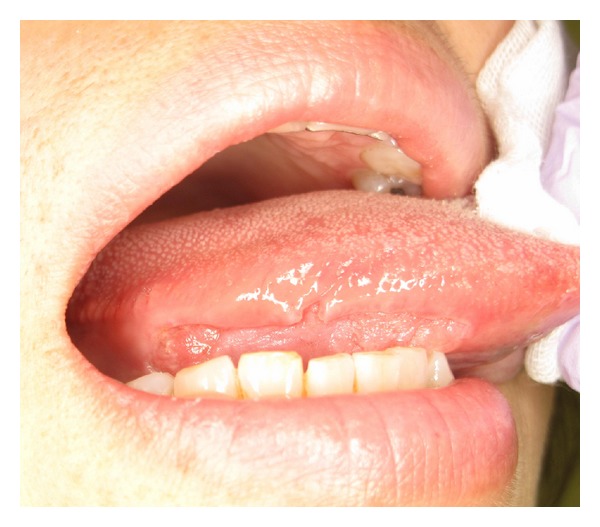
Examination of the oral cavity. An elastic, hard superficial mass with ulcer that measured approximately 40 × 20 mm is observed on the right side of the tongue.

**Figure 2:**
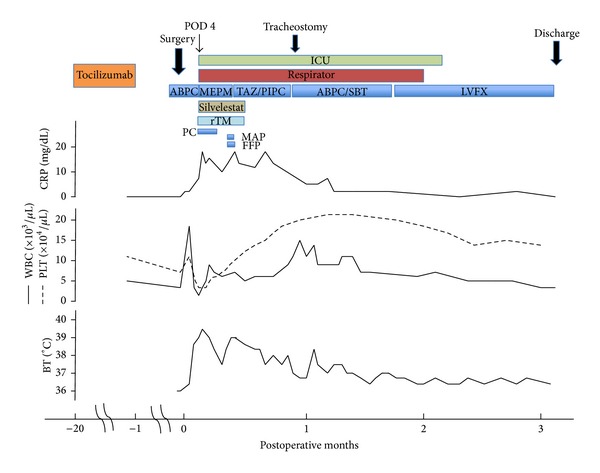
Clinical course. Measurements of CRP (C-reactive protein), WBC (white blood cells count), PLT (platelet count), and BT (body temperature) are taken before and after surgery. ICU (intensive care unit), PC (platelet concentrate), MAP (mannitol-adenine-phosphate), FFP (fresh frozen plasma), and rTM (recombinant thrombomodulin).

A 65-year-old woman came to the Department of Oral and Maxillofacial Surgery, University of Tsukuba Hospital, complaining of a painful mass on the right side of her tongue existing for 1 year. She had suffered from RA for 11 years and took Methotrexate for 3 years starting in 2008. Subsequently, TCZ was administered at a dose of 400 mg/month from November 2011 until December 2012, at which time the frequency of the dose was decreased to every 2 months. The last infusion of TCZ was about 1 month before the surgery. She had no rheumatoid lung. The social and family histories were unremarkable. Examination of the oral cavity showed an elastic hard mass on the right side of the tongue measuring approximately 40 × 20 mm (Figure 1). Biopsy of the tongue indicated that the mass was a well-differentiated squamous cell carcinoma. The regional lymph nodes were normal, and a chest radiograph disclosed no metastasis or occult disease. A partial glossectomy and supraomohyoid neck dissection were performed under general anesthesia without any complications as a diagnosis of tongue cancer (T2N0M0). The surgery lasted 4 hours and 32 minutes, and the blood loss was 110 mL. The nasal intubation tube was removed after the patient was wakened in the operating room. The patient's postsurgical clinical course is shown in Figure 2.

**Figure 3:**
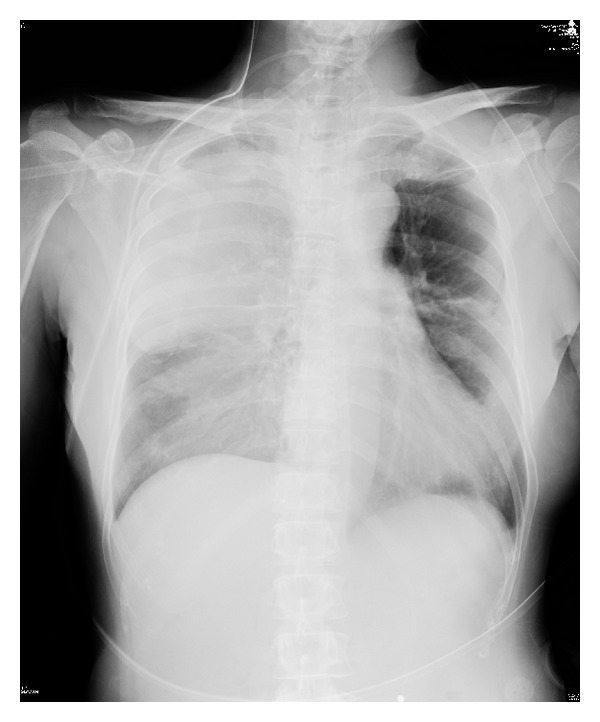
Chest X-ray (POD 4). The chest X-ray reveals a cloudy right lung and severe consolidation in the upper lobe of the lung.

**Figure 4:**
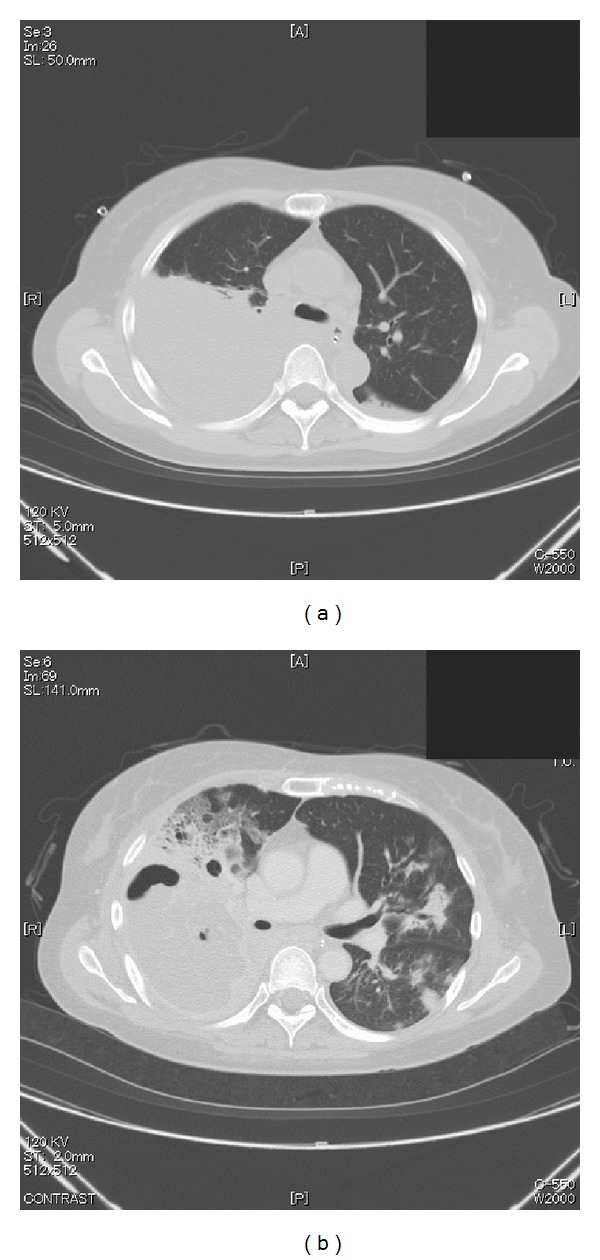
(a) Chest CT (POD 4). The scan shows a broad infiltrating shadow with air bronchogram mainly in the dorsal portion of the right lung, leading to a diagnosis of pulmonary suppuration. (b) Chest CT (POD 19). Two weeks later, the broad infiltrating shadow has expanded and shows storage of liquid. Both lungs have ground glass opacity and contain a maculate infiltrating shadow.

**Figure 5:**
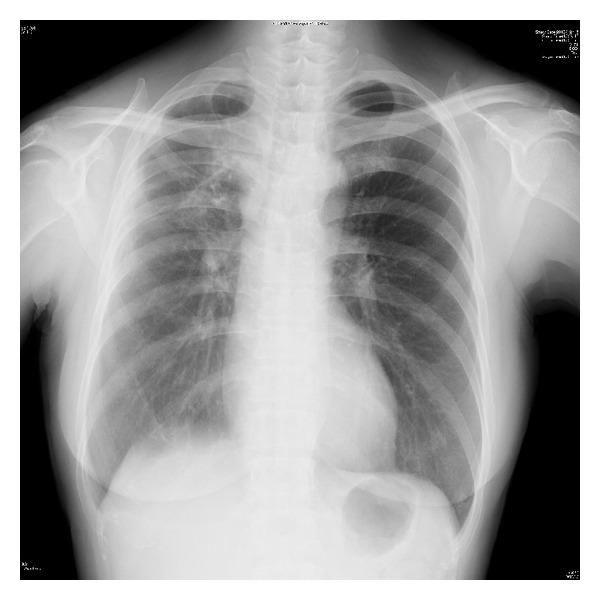
Chest X-ray (PO 4 months). A chest X-ray reveals an improved clear field in the right lung.

On postoperative day (POD) 1, the white blood cells (WBC) count rose to 18.5 × 10^3^/*μ*L and the neutrophil count rose to 17.2 × 10^3^/*μ*L, whereas the concentration of C-reactive protein (CRP) was 0.74 mg/dL. There was a significant discrepancy between the pre- and postoperative numbers for WBC and CRP. The vital signs on POD 2 were as follows: blood pressure 145/72 mmHg, pulse rate 102/min, respiratory rate 25/min, and body temperature (BT) 38.5°C. The lab data showed a WBC content of 3.6 × 10^3^/*μ*L and a CRP level of 1.0 mg/dL. These results led to a diagnosis of SIRS. SIRS criterion is presenting two or more of the following symptoms: (1) BT > 38°C or <36°C, (2) pulse rate > 90/min, (3) respiratory rate > 20/min or PaCO_2_ < 32 mmHg, and (4) WBC > 12.0 × 10^3^/*μ*L or <4.0 × 10^3^/*μ*L [8]. On POD 3, the patient's BT rose to 39.2°C, and she complained of cough and dyspnea, while the lab data indicated the following: platelet count (PLT) 46 × 10^3^/*μ*L; WBC 1.6 × 10^3^/*μ*L (Seg 2.0%, Band 47.0%); and CRP 8.6 mg/dL. The aspiration pneumonia had rapidly worsened. The patient was moved to an intensive care unit (ICU), a tracheal intubation was performed, and she was placed on a ventilator (F_*I*_O_2_ 0.6, PEEP 5 cm H_2_O) on POD 4. The arterial blood gas analysis revealed hypoxemia (pH 7.425, PaO_2_ 40.5 Torr, PaCO_2_ 65.1 Torr). From POD 4 to 13, the patient received recombinant thrombomodulin to treat the disseminated intravascular coagulation (PLT 9 × 10^3^/*μ*L, PT ratio 1.36, FDP 12.1 *μ*g/mL) and sivelestat for acute respiratory distress syndrome. Platelet concentrate was infused for a total of 40 U over 3 days. A chest X-ray revealed both a cloudy right lung and a severe consolidation in the upper lobe of the lung (Figure 3). A CT scan of the chest on POD 4 revealed a broad infiltrating shadow with air bronchogram mainly on the dorsal side of the right lung (Figure 4(a)). By POD 19, the infiltrating shadow had expanded and showed evidence of liquid storage. The follow-up CT scan taken at this time showed both lungs to have ground glass opacity and a maculate infiltrating shadow (Figure 4(b)). A diagnosis of pulmonary suppuration was made. Microorganism cultures of sputum detected* Klebsiella pneumonia*. The antibiotic regimen was changed from ampicillin (ABPC) to meropenem (MEPM) until POD 10 and then to tazobactam/piperacillin (TAZ/PIPC) until POD 21. The CRP value fell to the normal range at about PO 1 month, at which time extubation was attempted. However, because the patient's respiratory capacity was still poor, a tracheostomy was performed and another month of mechanical ventilation was added to her follow-up care. ABPC/sulbactam (SBT) was administered for about 1 month starting at PO 1 month, and the patient's BT resolved to less than 37°C. Thereafter, the patient was weaned from the ventilator and was moved from the ICU to a normal ward at about PO 2 months. She was discharged from the hospital at about PO 3 months. A chest X-ray revealed an improved clear field in the right lung (Figure 5) and the patient's respiratory function resolved. While the intraoral healing of the wound was slower than normal, there was no infection at the site of surgery, and the clinical course of the tongue cancer remained uneventful, without any recurrence or metastasis to the neck.

## 3. Discussion

In a clinical trial of IL-6 in patients with malignancies, IL-6 was found to induce fever, chills, and general malaise [9]. Grave adverse events associated with the use of TCZ include serious infections that may lead to hospitalization or death, gastrointestinal perforation, and hypersensitivity reactions including anaphylaxis [2]. The most common adverse events reported in clinical studies were upper respiratory tract infections, nasopharyngitis, headache, high blood pressure, and increased liver enzymes [2]. The rate of serious infections was 3.6 events per 100 patient-years, but the overall rate of fatal infections was low (0.13 events per 100 patient-years) [10].

Pneumonia is a common problem in HANC surgical patients. An increased risk of developing pneumonia is associated with advanced comorbidity, weight loss, and major surgical procedures, a heightened chance of infectious pneumonia is associated with chronic pulmonary disease, and an increased risk of aspiration pneumonia is associated with dysphagia [4]. Although only 4 accounts of organizing pneumonia induced by TCZ have been reported [5–7], no case of aspiration pneumonia after HANC surgery has been described as yet. To our knowledge, this is the first report in which an RA patient using TCZ, who underwent surgery for tongue cancer, subsequently developed severe pulmonary suppuration from aspiration pneumonia.

It is well recognized that the excessive production of proinflammatory cytokines such as TNF-*α*, IL-1*β*, IL-6, and IL-8 by immune-competent cells can induce SIRS and that these cytokines can also play an important role in the development of acute respiratory distress syndrome or multiple organ dysfunction syndrome [11, 12]. The excessive production of proinflammatory cytokines activates neutrophils, the coagulation system, and other mediator cascades, resulting in organ dysfunction. Moreover, blood cytokine levels begin to rise prior to the onset of organ dysfunction [11]. IL-6 induces acute-phase proteins like CRP, fibrinogen, *α*1-antitrypsin, and serum amyloid protein A in hepatocytes [2, 13]. In our patient, TCZ suppressed IL-6 as well as the early inflammatory symptoms of aspiration pneumonia. A slight increase in CRP (1.0 mg/dL) was observed in the laboratory findings on POD 2, accompanied by a high fever, but these levels quickly rose to 17.1 mg/dL following progression of the aspiration pneumonia on POD 4, suggesting that TCZ may have suppressed the early inflammatory reaction, leading to severe organ dysfunction.

A recent report investigating the relationship between TCZ treatment and joint surgery outcomes found no complications due to infection or delays in wound healing [13]. On the other hand, the guidelines for TCZ use published in 2009 mention that wound healing might be delayed under TCZ therapy. Moreover, these guidelines advise that surgery should be postponed in patients undergoing TCZ treatment until the concentration of the drug in the peripheral blood has fallen, deferring surgery until at least 14 days after the last infusion [14]. In the present case, although TCZ was last administered about 1 month before surgery and there was no infection at the surgical site, the intraoral wound healing was delayed more than expected. An earlier study concluded that TCZ suppressed fever and increased the level of CRP after surgery, whereas it found no influence of the drug on the number of leukocytes before and after surgery [13]. In the current case, the WBC count was high, but the CRP concentration was normal on POD1. Although the WBC and CRP levels were not remarkable, the BT rose on POD 2. The CRP changed later, which was in agreement with the previous report on TCZ-treated patients after surgery [12]. Since TCZ inhibits IL-6's actions, it is possible that these early symptoms of pneumonia were masked by the TCZ treatment.

Because the anti-inflammatory effect of TCZ may mask the typical symptoms and signs of infection and the possible conversion to severe infection-induced SIRS, surgeons need to be aware of the potential for hidden infections after surgery. This report provides rheumatologists and surgeons with useful information for the treatment and follow-up of rheumatology patients under TCZ treatment.
